# mRNA therapy: a precise and efficient approach for disease treatment

**DOI:** 10.1093/lifemedi/lnae035

**Published:** 2024-09-14

**Authors:** Jia-Yi Zhou, Ying Yang

**Affiliations:** China National Center for Bioinformation, Beijing 100101, China; Beijing Institute of Genomics, Chinese Academy of Sciences, Beijing 100101, China; China National Center for Bioinformation, Beijing 100101, China; Beijing Institute of Genomics, Chinese Academy of Sciences, Beijing 100101, China; Sino-Danish College, University of Chinese Academy of Sciences, Beijing 101408, China; Institute for Stem Cell and Regeneration, Chinese Academy of Sciences, Beijing 100101, China

**Figure 1.**
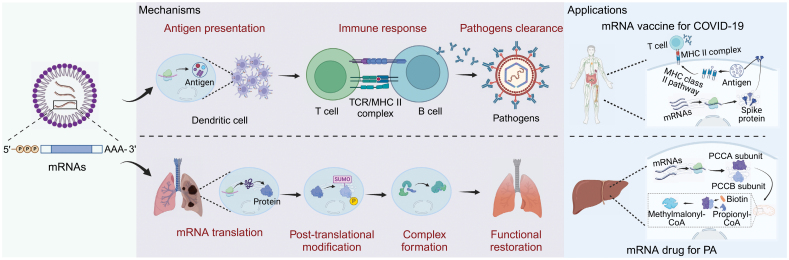
**Graphical overview of key mechanisms and applications of mRNA therapy.** The fundamental concept of mRNA vaccines revolves around the utilization of mRNA synthesized *in vitro* to produce specific antigens within the body. These antigens, in turn, prompt the immune system to respond and confer protection against various diseases. This strategy primarily encompasses three distinct stages: antigen presentation, immune response, and pathogen clearance. On the other hand, mRNA drugs involve the delivery of synthesized mRNA to target cells, enabling the production of proteins through translation, post-transcriptional modification, and complex formation processes. Created with BioRender.

The field of messenger RNA (mRNA) therapeutics is currently at the forefront of medical research, with numerous clinical trials actively exploring its potentials. Pioneering experiments in this field were conducted by Wolff et al., who were the first to inject synthetic mRNA into the skeletal muscle of mice [1]. They observed that the mRNA was successfully translated into the corresponding protein *in vivo*, making a significant milestone in RNA-based drug development. Subsequently, technological advancements, including the reduction of immunogenicity in exogenous mRNA through chemical modifications and the optimization of delivery systems to enhance mRNA delivery efficiency, have demonstrated the application value of mRNA therapy in disease prevention and treatment. This includes its use in infectious disease vaccines, tumor immunotherapy, and protein replacement therapy. Since mRNA remains in the cytoplasm for translation rather than entering the cell nucleus to perform functions, the risk of genomic integration is minimized, conferring advantages to mRNA therapy such as high controllability and reduced risk. Furthermore, mRNA therapy has the ability to deliver multiple mRNAs for the translation of multimeric proteins. Depending on different principles, mRNA therapy is primarily categorized into two aspects. One aspect involves mRNA drugs that specifically deliver synthesized mRNA to target cells, thereby leading to the synthesis of desired proteins, and their subsequent functional roles. The other aspect is mRNA vaccines, which introduce mRNA into the body, allowing for the translation of antigen proteins, and the triggering of an immune response [2] (Fig. 1).

A recent study published in *Nature* reported the outcomes of the first clinical trial utilizing mRNA therapy as a replacement therapy for intracellular proteins [3]. Given that synthetic mRNA closely resembles natural mRNA, patients are able to produce therapeutic proteins *in vivo*, eliminating the intricate process of recombinant protein manufacturing *in vitro*. This trial employed mRNA-3927 to treat patients with propionic acidemia (PA) through genetic testing, assessing both the safety and effectiveness of this treatment. PA is an autosomal recessive organic acidemia caused by mutations in propionyl-coenzyme A (CoA) carboxylase (PCC) α or β subunit (*PCCA* or *PCCB*) genes. Previous research conducted on mouse model has demonstrated that mRNA-3927 exhibits a rapid onset of action when compared to traditional drugs [4]. Notably, expression levels of PCC protein subunits and enzymes increased significantly within 6 hours of administration, effectively restoring physiological processes of propionic acid metabolism in the mouse liver. The mRNA-3927 utilized in this recent clinical trial comprises two mRNAs: one encoding the normal human PCCA subunit and another encoding PCCB subunit. These mRNAs are delivered into the cytoplasm using lipid nanoparticles (LNPs) and subsequently translated into the PCCA and PCCB subunits, forming the PCC enzyme complex. This complex is ultimately localized in the mitochondria to play crucial roles in the propionate metabolic pathway. This human clinical trial involved 16 patients with PA across 5 dose groups, 12 of whom completed the dose optimization study and participated in the extension study. Throughout the treatment course, which involves several prolonged injections, 15 patients experienced adverse treatment effects, including fever, vomiting, and diarrhea. Eight patients experienced serious adverse effects, but none were dose-limiting. Additionally, although individual patients initially tested positive for anti-polyethylene glycol (PEG) antibodies during treatment, these levels gradually diminished and returned to normal or became negative in subsequent sessions, indicating that the mRNA-3927 treatment does not elicit an abnormal immune response. Finally, the pharmacokinetic parameters of *PCCB* mRNA following both single and repeated doses are currently under investigation. These parameters will be crucial in determining the optimal therapeutic dose and subsequently assessing the effectiveness of this therapy through another round of clinical trials. Notably, compared to their pretreatment state, patients exhibited a decrease in disease biomarkers and a 70% reduction in the risk of metabolic decompensation events following treatment. This suggests that mRNA-3927 is effectively transported into hepatocytes through intravenous infusion and undergoes translation and post-translational modifications, resulting in the production of functional PCC enzymes.

Despite the patients achieving remission following mRNA-3927 treatment, statistical significance of the results remained elusive due to the limited number of participants and the lack of a control group. Additionally, the trial cohort was primarily composed of whites and Asian individuals, necessitating further expansion of the sample size to assess the effectiveness of the treatment across diverse populations. Nonetheless, this report is important in demonstrating the potential clinical worth of mRNA therapy for genetic diseases and addressing researchers’ concerns regarding the immune response elicited by long-term, repeated administrations of high-dosage of mRNA-containing LNP.

Another type of mRNA therapy is based on the principle of translating specific mRNAs into antigens, thereby activating the host’s autoimmune. In contrast to previous mRNA therapies, this approach requires the production of only a minimal amount of protein. Subsequently, the human immune system amplifies immune signals through cell- and antibody-mediated immune responses. This therapeutic approach finds widespread applications in prophylactic vaccines and tumor therapy. mRNA vaccines introduce mRNA fragments encoding pathogen-specific proteins into human cells. These proteins are first translated and then trigger host-specific immune responses. This method elicits a specific immune response without the need for exposure to the intact virus, thereby preventing disease in a safe manner. Currently, mRNA vaccines have proven successful in a variety of disease prevention applications, including severe acute respiratory syndrome coronavirus 2 (SARS-COV-2) and Zika virus. The prevention of infectious diseases through mRNA vaccines is characterized by rapid development, simplicity, and fast production. Furthermore, mRNA vaccine makes use of the ability of mRNA to transmit genetic information and its inherent immunostimulatory activity to aid in tumor therapy. Researchers screen and analyze tumor-related antigens and create vaccines based on corresponding mRNA sequences. These vaccines prompt the patient’s own cells to produce antigen recognizers, activate immune responses, accurately target tumors, and enhance the efficiency of killing tumor cells.

A variety of vaccines targeting different cancers are currently undergoing clinical testing. For example, mRNA-4157 was designed based on the unique mutational signature of patient’s tumor DNA sequence to create personalized vaccines, which can promote the generation of specific T-cell anti-tumor responses [5]. Of the 13 patients treated with mRNA-4157, 11 remained disease-free for up to 75 weeks. The accurate setting and high controllability of the mRNA sequence enables a more tailored therapeutic approach for specific diseases and individuals, thereby augmenting both safety and reliability. Additionally, the short lifespan of mRNA makes it less likely to cause adverse reactions, while nucleoside modifications, including pseudouridine, effectively enhance mRNA stability, translational capacity, and reduce immunogenicity. Furthermore, when combined with traditional drugs, mRNA therapy provides a more potent means of disease treatment. For example, the combined recipe of mRNA-4157 and the programmed death receptor-1 (PD-1) inhibitor Keytruda has demonstrated the generation of neoantigen-specific T-cell responses, effectively suppressing cancer development [5]. Although mRNA vaccines offer precise and efficient tumor eradication through targeted therapy, the complexity of genetic variations and varied expression patterns across diverse cancers necessitates the development of precise, personalized mRNA vaccines.

Despite its safety, programmability, and flexibility, mRNA therapy faces significant hurdles stemming from its inherent characteristics and technical limitations, hindering widespread applications. Notably, the susceptibility of mRNA to degradation poses a challenge in ensuring its efficient *in vivo* delivery. Although intravenous injection readily targets mRNA therapies to the liver, achieving effective delivery to other solid organs remains more challenging. To address these limitations, researchers are exploring innovative delivery methods, including the utilization of inorganic nanomaterials. Furthermore, modifying specific nucleosides of mRNAs to enhance translation efficiency is a promising approach to boost the efficiency of mRNA therapy. While the current focus of mRNA therapy is primarily on disease prevention and treatment, future research exploring its potential in tissue regeneration and aging delay holds great significance.
